# Caffeinated Coffee Consumption and Atrial Arrhythmia Recurrence After Pulmonary Vein Isolation

**DOI:** 10.3390/jcm15155841

**Published:** 2026-07-26

**Authors:** Patricia Hägele, Viola Adam, Paloma Biehler, Stephanie Löbig, Andrei Pinchuk, Christian Waechter, Dominik Buckert, Sebastian Weyand, Peter Seizer

**Affiliations:** 1 Department of Cardiology and Angiology, Medical Clinic II, Ostalb Hospital Aalen, 73430 Aalen, Germany; patricia.haegele@kliniken-ostalb.de (P.H.); viola.adam@kliniken-ostalb.de (V.A.); paloma.biehler@kliniken-ostalb.de (P.B.); stephanie.loebig@kliniken-ostalb.de (S.L.); andrei.pinchuk@kliniken-ostalb.de (A.P.); 2 Department of Cardiology III: Adult Congenital Heart Disease and Valvular Heart Disease, University Hospital Münster, 48149 Münster, Germany; c.waechter@uni-muenster.de; 3 Department of Cardiology, Angiology, Pneumology and Internal Intensive Care Medicine, Ulm University Hospital, 89081 Ulm, Germany; 4 Faculty of Medicine, Ulm University, 89081 Ulm, Germany

**Keywords:** atrial fibrillation, pulmonary vein isolation, coffee consumption, caffeine, catheter ablation

## Abstract

**Background:** The impact of caffeinated coffee consumption on atrial fibrillation (AF) recurrence after pulmonary vein isolation (PVI) remains uncertain, and clinical recommendations regarding caffeine intake are inconsistent. **Methods:** This retrospective observational cohort study included consecutive patients undergoing first-time PVI using a high-power short-duration technique between 2019 and 2023. Patients were stratified according to self-reported daily coffee consumption into a high-consumption group (≥2 cups/day) and a low-consumption group (0–1 cup/day). The primary endpoint was recurrence of atrial arrhythmia within 12 months after PVI, excluding a 90-day blanking period. Multivariable logistic regression was performed to assess the association between coffee consumption and arrhythmia recurrence. **Results:** A total of 199 patients were included, of whom 124 (62.3%) reported high coffee consumption and 75 (37.7%) low consumption. Baseline characteristics and procedural parameters were comparable between groups. At 12 months, atrial arrhythmia recurrence occurred in 24 patients (19.4%) in the high-consumption group and 25 patients (33.3%) in the low-consumption group (*p* = 0.03). In multivariable analysis, higher coffee consumption was independently associated with a lower risk of recurrence (adjusted OR 0.47, 95% CI 0.23–0.95; *p* = 0.036). **Conclusions:** In patients undergoing first-time PVI, higher habitual consumption of caffeinated coffee was associated with a lower risk of atrial arrhythmia recurrence within one year. These findings suggest that moderate coffee consumption may be safely permitted and need not be routinely restricted in this setting. Further prospective studies are warranted to clarify whether this association is causal.

## 1. Introduction

Atrial fibrillation (AF) is the most common sustained cardiac arrhythmia worldwide, with an estimated lifetime risk of approximately 25% [1]. It is associated with a substantial increase in the risk of stroke, heart failure, and all-cause mortality, representing a major public health burden [2].

Pulmonary vein isolation (PVI) is an established cornerstone in the treatment of symptomatic AF and has been shown to improve quality of life compared with medical therapy alone [3]. However, despite advances in ablation techniques, arrhythmia recurrence remains frequent, occurring in approximately 20–40% of patients during follow-up [4,5]. This underscores the need to better understand potentially modifiable lifestyle factors that may influence post-ablation outcomes.

Coffee is one of the most widely consumed beverages worldwide and the primary source of caffeine intake in many populations. The relationship between coffee consumption and AF remains controversial. While some patients report coffee as a trigger for arrhythmic episodes, observational studies have suggested neutral or even beneficial associations with cardiovascular outcomes, including reduced cardiovascular and all-cause mortality [6,7,8]. These effects have been attributed not only to caffeine but also to other bioactive compounds such as polyphenols and chlorogenic acids, which may exert antioxidant, anti-inflammatory, and metabolic effects [9,10,11]. In addition, habitual coffee consumption has been associated with improved glucose metabolism and a lower risk of type 2 diabetes, an established risk factor for AF [12].

Data on the impact of coffee consumption on AF recurrence after catheter ablation are limited. Recently, the randomized DECAF trial suggested that moderate consumption of caffeinated coffee was associated with a lower recurrence of atrial arrhythmias following cardioversion compared with abstinence [13]. However, evidence in patients undergoing contemporary ablation strategies remains scarce.

Therefore, the present study aimed to evaluate the association between habitual caffeinated coffee consumption and the recurrence of atrial arrhythmias within one year after first-time PVI in patients with AF.

## 2. Materials and Methods

### 2.1. Study Population

This retrospective observational cohort study included consecutive patients with atrial fibrillation (AF) who underwent first-time pulmonary vein isolation (PVI) with a high-power short-duration (HPSD) technique at Ostalb-Klinikum Aalen between 2019 and 2023. Patients with prior PVI procedures or incomplete follow-up data were excluded.

Patients were stratified according to self-reported habitual daily coffee consumption assessed at baseline. During the routine pre-procedural medical history, patients were asked about their average daily intake of caffeinated coffee. When patients reported different cup sizes, coffee intake was clarified and documented as the estimated equivalent number of standard-sized cups consumed per day. Patients were categorized into a high-consumption group (≥2 cups/day) and a low-consumption group (0–1 cup/day). This categorization was chosen to distinguish patients with little or no habitual coffee intake from those with more regular coffee consumption. The selected cut-off was not intended to represent an established biological threshold. Information regarding coffee preparation method (e.g., espresso or filtered coffee) was not routinely documented and was therefore unavailable for analysis. Smoking status and alcohol consumption were likewise documented during routine baseline assessment. Current nicotine use was defined as current smoking at the time of the procedure. High-risk alcohol consumption was defined as a daily intake exceeding 20 g of alcohol in women and 30 g in men.

The study was approved by the local ethics committee of the Landesärztekammer Baden-Württemberg. Written informed consent for the ablation procedure and routine post-procedural care was obtained from all patients.

### 2.2. Ablation Procedure

All PVI procedures were performed according to the institutional “Aalen protocol” as previously described [14,15].

All patients received oral anticoagulation for at least three weeks prior to the procedure and for a minimum of three months thereafter, irrespective of their CHA_2_DS_2_-VA score. Direct oral anticoagulants (DOACs) were continued uninterrupted, except for the morning dose on the day of the procedure, which was administered four hours after ablation in the absence of bleeding complications. Vitamin K antagonists were maintained with a target international normalized ratio (INR) of 2.0–3.0. Procedures were performed under deep intravenous sedation in spontaneously breathing patients. Intravenous heparin was administered during the procedure to maintain an activated clotting time between 300 and 400 s.

Double transseptal access was established. Electroanatomical mapping was performed using either the CARTO system (Biosense Webster, Diamond Bar, CA, USA) or the EnsiteX system (Abbott Laboratories, Chicago, IL, USA). High-resolution mapping catheters (e.g., Pentaray (Biosense Webster, Diamond Bar, CA, USA) or Advisor HD Grid (Abbott Laboratories, Chicago, IL, USA)) and open-irrigated, contact force-sensing ablation catheters (e.g., Thermocool SmartTouch (Biosense Webster, Diamond Bar, CA, USA) or Tacticath Quartz (Abbott Laboratories, Chicago, IL, USA)) were used. A detailed electroanatomical map of the left atrium (LA) and pulmonary veins (PV) was created in all patients. Low-voltage areas (<0.5 mV) were quantified using the integrated mapping software and expressed as a percentage of total LA surface area. Mapping was preferentially performed in sinus rhythm. If patients presented in AF, electrical cardioversion was performed.

Wide antral circumferential point-by-point ablation was performed to achieve PVI using radiofrequency energy of 50 W for 15 s per point at the anterior wall and 10 s per point at the posterior wall. The targeted contact force ranged from 3 to 20 g. Esophageal temperature monitoring was routinely performed.

Pulmonary vein isolation was confirmed by demonstration of both entrance and exit block, including assessment of the carina between ipsilateral veins. Adenosine (12 mg intravenously) was administered to unmask dormant conduction. Additional ablation was performed in case of residual conduction gaps. If AF persisted after ablation, electrical cardioversion was performed to restore sinus rhythm. After the procedure, patients were monitored with continuous rhythm surveillance for at least 24 h.

### 2.3. Follow-Up

Following the procedure, patients were followed according to institutional standard protocols. Routine follow-up included 24 h Holter monitoring at 6 and 12 months after PVI, as well as additional evaluations in case of symptoms suggestive of arrhythmia recurrence. Patients were instructed to contact the study center in case of recurrent arrhythmia or symptoms related to AF or heart failure. All available clinical data, including outpatient visits and hospital records, were reviewed to identify arrhythmia recurrences. Procedure-related complications were assessed during the index hospitalization and within 30 days after PVI based on all available clinical records.

### 2.4. Endpoint

The primary endpoint was recurrence of atrial arrhythmia within 1 year after PVI, defined as any episode of AF or AT lasting more than 30 s and documented by 12-lead electrocardiography or Holter monitoring. A blanking period of 90 days after the procedure was applied. Recurrences occurring within this period were not considered as endpoint events. Only recurrences occurring after the blanking period and within the first year of follow-up were included in the primary endpoint analysis.

Safety endpoints included procedure-related complications, namely procedure-related death, atrio-esophageal fistula, stroke or transient ischemic attack, pericardial tamponade requiring intervention, postinterventional pericarditis or small pericardial effusion, hemothorax, severe air embolism with ST-segment elevation, new-onset renal failure requiring dialysis, vascular access complications requiring or not requiring intervention, and acute hypersensitivity reactions to contrast agents.

### 2.5. Statistical Analysis

Categorical variables are presented as counts and percentages and were compared using the chi-squared test or Fisher’s exact test, as appropriate. Continuous variables are expressed as mean ± standard deviation and were compared using the unpaired Student’s t-test or the Mann–Whitney U test, depending on data distribution. Normality was assessed using the Shapiro–Wilk test. Event-free survival, defined as the time from PVI to the first documented episode of AF or AT, was analyzed using the Kaplan–Meier method and compared between groups using the log-rank test. Time-to-event analyses were considered descriptive. Because the primary comparative analysis focused on atrial arrhythmia recurrence within 1 year after PVI as a fixed binary outcome, multivariable logistic regression was used to identify independent associations with recurrence. Given the limited number of events, the model was restricted to a small number of clinically relevant covariates to avoid overfitting. To assess the robustness of the categorical analysis, coffee intake was additionally modeled as a continuous variable, and adjusted predicted probabilities were derived from the multivariable logistic regression model. Results are reported as odds ratios (OR) with 95% confidence intervals (CI). A two-sided *p*-value < 0.05 was considered statistically significant. All statistical analyses were performed using R 4.5.1 (R Foundation for Statistical Computing, Vienna, Austria), Microsoft Excel 2021 (Microsoft Corporation, Redmond, WA, USA), and GraphPad Prism 11 (GraphPad Software, Inc., San Diego, CA, USA). A graphical abstract was created using FigureLabs (available at https://www.figurelabs.ai (accessed on 19 April 2026)).

## 3. Results

### 3.1. Baseline Characteristics

A total of 199 consecutive patients undergoing first-time PVI using HPSD technique between 2019 and 2023 were included in the analysis. All included patients had completed the 12-month follow-up. Among these, 124 patients (62.3%) reported high coffee consumption (≥2 cups/day), whereas 75 patients (37.7%) reported low coffee consumption (0–1 cup/day). Overall, baseline clinical characteristics were comparable between groups (Table 1). There were no significant differences with regard to age, sex distribution, body mass index, AF type, or CHA_2_DS_2_-VA score (all *p* > 0.05). Likewise, echocardiographic parameters, including left ventricular ejection fraction, left atrial diameter, and the extent of left atrial low-voltage area, were similar between groups. Comorbidities and baseline medication use also did not differ significantly. As expected, mean daily coffee intake was significantly higher in the high-consumption group (3.26 ± 1.26 vs. 0.53 ± 0.50 cups/day, *p* < 0.01; Table 1).

### 3.2. Procedural Characteristics and Clinical Outcomes

**Figure 1:**
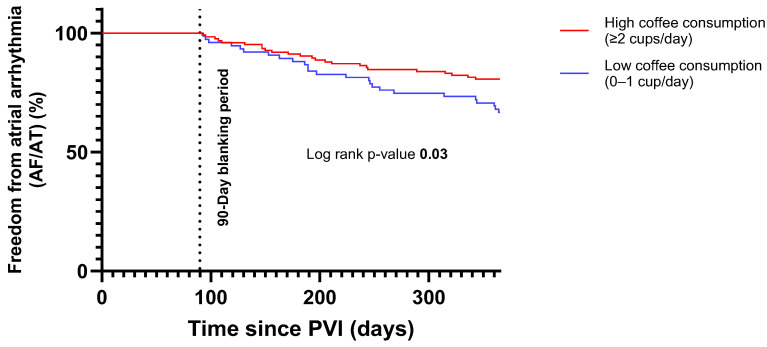
Time-to-event analysis showing freedom from atrial arrhythmia (AF/AT) following pulmonary vein isolation, stratified by coffee consumption. Kaplan–Meier curves demonstrate freedom from atrial arrhythmia after pulmonary vein isolation in patients with high (≥2 cups/day) versus low (0–1 cup/day) coffee consumption. The 90-day post-procedural blanking period is indicated. Differences between groups were assessed using the log-rank test.

Procedural characteristics were similar between patients with high and low coffee consumption (Table 2). Acute procedural success, defined as pulmonary vein entrance and exit block, was achieved in all patients in both groups (100% vs. 100%; *p* = 1.0). There were no significant differences in procedural duration, fluoroscopy time, or radiation exposure (all *p* > 0.05). At 1-year follow-up, atrial arrhythmia recurrence occurred in 24 patients (19.4%) in the high-coffee-consumption group compared with 25 patients (33.3%) in the low consumption group (*p* = 0.03; Table 2). Time-to-event analysis demonstrated a significantly higher freedom from atrial arrhythmia in patients with high coffee consumption (log-rank *p* = 0.03; Figure 1).

### 3.3. Safety Outcomes

Overall, safety outcomes were comparable between groups (Table 3). The incidence of complications was low and did not differ significantly between patients with high and low coffee consumption (4.0% vs. 4.0%; *p* = 1.0). No procedure-related deaths, atrio-esophageal fistulae, or strokes were observed in either group. The most frequent complication was postinterventional pericarditis or small pericardial effusion, which occurred at low rates in both groups. Vascular access complications were rare and did not require intervention in most cases.

### 3.4. Multivariable Analysis

**Figure 2:**
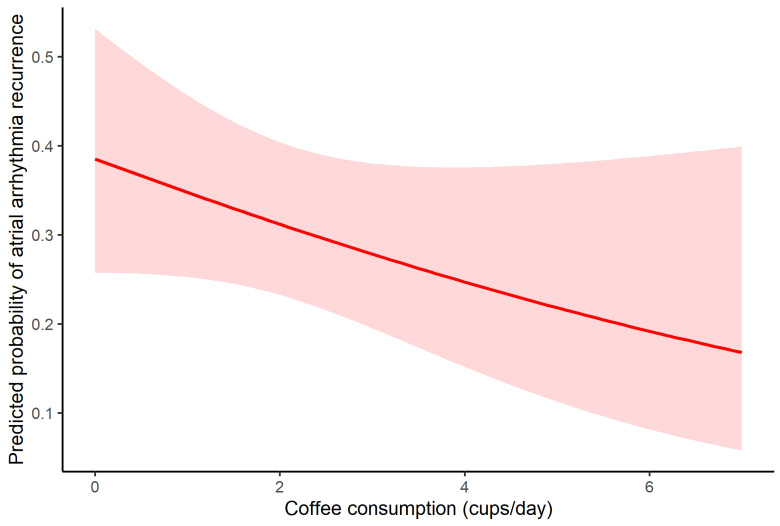
Adjusted probability of atrial arrhythmia recurrence according to coffee consumption. Predicted probabilities of atrial arrhythmia recurrence were derived from a multivariable logistic regression model including coffee consumption as a continuous variable and adjusted for age, type of atrial fibrillation, left ventricular ejection fraction, and left atrial diameter. The red line represents the adjusted predicted probability of atrial arrhythmia recurrence, and the shaded area represents the 95% confidence interval.

In multivariable logistic regression analysis, high coffee consumption was independently associated with a lower risk of atrial arrhythmia recurrence after PVI (adjusted OR 0.47, 95% CI 0.23–0.95; *p* = 0.036; Table 4). Among covariates, persistent atrial fibrillation was associated with an increased risk of recurrence (adjusted OR 2.46, 95% CI 1.06–6.33; *p* = 0.046), whereas age and left ventricular ejection fraction were not significantly associated with outcome. Left atrial diameter showed a trend toward higher recurrence risk (adjusted OR 1.05 per mm, 95% CI 1.00–1.10; *p* = 0.07). In a sensitivity analysis treating coffee consumption as a continuous variable, higher coffee intake showed a consistent inverse association with recurrence, although this did not reach statistical significance after adjustment (OR per cup/day 0.85, 95% CI 0.68–1.06; *p* = 0.154). The adjusted probability of atrial arrhythmia recurrence according to coffee consumption is illustrated in Figure 2.

## 4. Discussion

In this study of patients with AF undergoing pulmonary vein isolation, consumption of ≥2 cups of caffeinated coffee per day, compared with low or no intake (0–1 cups per day), was associated with a significantly lower recurrence of AF or atrial flutter within the first year after the procedure. These findings challenge the traditional assumption that coffee promotes cardiac arrhythmias and are consistent with prior observational data suggesting a neutral or potentially beneficial association between coffee consumption and cardiovascular outcomes, including AF [8,10].

### 4.1. Context and Interpretation

Previous observational studies have reported largely neutral or even beneficial associations between habitual coffee consumption and the risk of atrial fibrillation as well as broader cardiovascular outcomes [10,16]. However, findings across studies have not been entirely consistent [17] and concerns about potential proarrhythmic effects of caffeine persist in clinical practice. The present study extends this body of evidence by demonstrating a similar association in patients undergoing contemporary catheter ablation, a population in which data have been limited.

In addition, our findings are consistent with the recently published DECAF trial, which suggested a lower recurrence of atrial arrhythmias following cardioversion among patients consuming caffeinated coffee compared with abstinence [13]. While causal conclusions cannot be drawn from our observational analysis, our data support this hypothesis in a contemporary PVI population.

When coffee consumption was analyzed using the prespecified categorical cut-off (≥2 vs. 0–1 cups/day), the association remained significant, whereas the sensitivity analysis treating coffee intake as a continuous variable did not remain statistically significant after multivariable adjustment. Although only the categorical analysis was statistically significant, the point estimates were directionally consistent across both models. The lack of statistical significance in the continuous model may reflect limited statistical precision, as well as the assumption of a constant linear effect per additional cup of coffee in the continuous model. Consequently, the present data support an association at the prespecified cut-off but do not establish a linear dose–response relationship or demonstrate that two cups per day represent a biological threshold.

However, these findings should be interpreted with caution. As an observational study, residual confounding cannot be excluded, even though the association remained consistent after multivariable adjustment. In particular, moderate coffee consumption may reflect a generally healthier lifestyle, including differences in diet, physical activity, and socioeconomic factors. Therefore, coffee intake may act as a surrogate marker rather than a direct causal factor, and the observed association should be considered hypothesis-generating.

### 4.2. Potential Mechanisms

Several mechanisms may explain the observed association between habitual coffee consumption and a lower risk of atrial arrhythmia recurrence after pulmonary vein isolation. Importantly, recurrence after ablation is not solely driven by acute arrhythmic triggers but also by an underlying atrial substrate characterized by structural and electrical remodeling, including inflammation and fibrosis [18,19].

Caffeine acts as a non-selective antagonist of adenosine receptors, which are involved in atrial electrophysiology [20]. Since adenosine can shorten atrial refractoriness and facilitate arrhythmogenesis, attenuation of these effects by caffeine may reduce susceptibility to atrial arrhythmias [21]. In addition, coffee contains various bioactive compounds, including polyphenols and chlorogenic acids, which exert antioxidant and anti-inflammatory effects [10,22,23]. As inflammation and oxidative stress contribute to atrial remodeling, these mechanisms may favorably influence the atrial substrate and thereby reduce the likelihood of arrhythmia recurrence after PVI.

Experimental and clinical data suggest that caffeine does not consistently increase atrial ectopy or acute arrhythmia triggers [24]. This distinction between acute triggers and the underlying substrate may help explain the findings of the present study: while coffee consumption may have limited impact on trigger activity, it could modulate the atrial substrate and thereby contribute to improved rhythm stability following ablation.

Caffeine also affects the autonomic nervous system, which plays a central role in the initiation and maintenance of atrial fibrillation [25]. While acute intake may increase sympathetic activity, habitual consumption has been associated with adaptive responses, including attenuation of sympathetic effects [16,26]. This distinction between acute and chronic exposure may further explain the lack of a consistent proarrhythmic effect observed in clinical studies.

Overall, these mechanisms are likely to interact and may be modified by individual patient characteristics. Therefore, the observed association should be considered biologically plausible but not causal.

### 4.3. Clinical Implications

The findings of this study suggest that moderate consumption of caffeinated coffee may be safely permitted in patients with atrial fibrillation undergoing PVI. While a potential beneficial association cannot be excluded, these results do not support a routine recommendation for complete abstinence from coffee and suggest that such restrictions may not be necessary in all patients.

Consistent with this, current international AF guidelines, including the 2024 ESC and 2023 ACC/AHA recommendations, do not advocate routine restriction of caffeine or coffee consumption in patients with atrial fibrillation [27]. Instead, management should focus on established modifiable risk factors, including weight reduction, treatment of diabetes, promotion of physical activity, reduction in alcohol intake and management of sleep apnea [28].

Taken together, these findings support an individualized approach to lifestyle modification, taking into account patient-specific factors, habitual caffeine intake, and potential patient-reported triggers. In the context of post-ablation management, moderate coffee consumption may be considered as part of a personalized strategy rather than being uniformly restricted.

However, given the observational nature of the present study, these findings should not be interpreted as evidence for a causal benefit. Further prospective and randomized studies are required to determine whether coffee consumption directly influences arrhythmia recurrence after catheter ablation.

### 4.4. Limitations

This study has several limitations that should be considered when interpreting the results. First, the single-center design may limit the generalizability of the findings to other populations and clinical settings. Second, coffee consumption was self-reported at baseline, which may introduce recall bias and does not account for potential changes in intake over time. In addition, the categorization of coffee consumption may not fully capture dose–response relationships and does not account for variations in cup size, preparation methods or actual caffeine content. Third, residual confounding cannot be excluded. Although multivariable adjustment was performed, habitual coffee consumption may represent a marker of broader lifestyle characteristics. Unmeasured factors, including physical activity, dietary habits, sleep quality, socioeconomic status, and differences in comorbidities, may have influenced the observed associations. Finally, rhythm monitoring was based on routine clinical follow-up, including intermittent Holter recordings and symptom-driven evaluation. As a result, asymptomatic episodes of atrial arrhythmia may have been underdetected.

## 5. Conclusions

In this cohort of patients with AF undergoing first-time PVI, higher habitual consumption of caffeinated coffee (≥2 cups/day) was associated with a lower risk of atrial arrhythmia recurrence within one year. These findings challenge the common assumption that caffeine promotes arrhythmias and suggest that moderate coffee consumption does not confer an increased arrhythmic risk in this setting. While causality cannot be inferred from this observational analysis, routine restriction of coffee intake may not be necessary in all patients undergoing catheter ablation. Further prospective and randomized studies are needed to clarify whether coffee consumption directly influences arrhythmia recurrence after PVI.

## Figures and Tables

**Table 1:** Baseline Clinical Characteristics (*n* = 199).

Variable	High Coffee Consumption (≥2 Cups/Day), *n* = 124	Low Coffee Consumption (0–1 Cup/Day), *n* = 75	*p*-Value
Age, years	66.31 ± 11.17	66.20 ± 11.44	0.79
Male, *n* (%)	82 (66.13)	48 (64)	0.76
BMI, kg/m^2^	28.54 ± 5.72	28.85 ± 5.2	0.53
Paroxysmal AF, *n* (%)	43 (34.68)	20 (26.67)	0.24
Persistent AF, *n* (%)	81 (65.32)	56 (73.33)	0.24
CHA_2_DS_2_-VA score	2.04 ± 1.41	2.15 ± 1.4	0.64
LV ejection fraction, %	54.92 ± 9.64	52.47 ± 10.97	0.16
LA diameter, mm	44.58 ± 8.04	46.25 ± 6.38	0.26
LA low voltage area, %	22.59 ± 27.13	16.83 ± 23.79	0.18
Moderate or severe MR	37 (29.84)	26 (34.67)	0.48
HFrEF, *n* (%)	13 (10.48)	11 (14.67)	0.38
Chronic kidney disease, *n* (%)	17 (13.71)	12 (16)	0.66
Coronary artery disease, *n* (%)	27 (21.77)	17 (22.67)	0.88
Hypertension, *n* (%)	74 (59.68)	45 (60)	0.96
Diabetes mellitus, *n* (%)	16 (12.9)	12 (16)	0.54
Prior Stroke/TIA, *n* (%)	4 (3.23)	5 (6.67)	0.30
Coffee consumption, cups/day	3.26 ± 1.26	0.53 ± 0.5	<0.01 *
Current nicotine use, *n* (%)	18 (14.52)	10 (13.33)	0.82
High-risk alcohol use, *n* (%)	24 (19.35)	12 (16)	0.46
Current Medication			
Beta-blockers	105 (84.68)	69 (92)	0.13
Amiodarone	11 (8.87)	9 (12)	0.48
Other antiarrhythmic drugs	4 (3.23)	2 (2.67)	1.0
Direct oral anticoagulant	122 (98.39)	74 (98.67)	1.0
Vitamin K antagonist	2 (1.61)	1 (1.33)	1.0

Data are presented as mean ± standard deviation or *n* (%). * *p* < 0.05 was considered statistically significant. Abbreviations: BMI, body mass index; AF, atrial fibrillation; LV, left ventricle; LA, left atrium; HFrEF, Heart failure with reduced ejection fraction; TIA, transient ischemic attack.

**Table 2:** Procedural parameters of pulmonary vein isolation (*n* = 199).

Variable	High Coffee Consumption (≥2 Cups/Day), *n* = 124	Low Coffee Consumption (0–1 Cup/Day), *n* = 75	*p*-Value
PV entrance and exit block, *n* (%)	124 (100)	75 (100)	1.0
Procedural duration (min)	106.01 ± 26.63	105.01 ± 28.85	0.81
Dose area product (cGy × cm^2^)	1233 ± 1029	1199 ± 1002	0.82
Fluoroscopy time (min)	12.5 ± 6.12	11.84 ± 6.48	0.43
Safety End Point, *n* (%)	5 (4.03)	3 (4)	1.0
Recurrence of AF/AT within 1 year after PVI	24 (19.35)	25 (33.33)	0.03 *

Data are presented as mean ± standard deviation or *n* (%). * *p* < 0.05 was considered statistically significant. Abbreviations: PV, pulmonary veins; PVI, pulmonary vein isolation; AF, atrial fibrillation; AT, atrial tachycardia.

**Table 3:** Safety End Points.

	High Coffee Consumption (≥2 Cups/Day), *n* = 124	Low Coffee Consumption (0–1 Cup/Day), *n* = 75
**Total**	**5 (4.03)**	**3 (4)**
Procedure-related deaths	0 (0)	0 (0)
Atrio-esophageal fistulae	0 (0)	0 (0)
Procedure-related strokes or TIAs	0 (0)	0 (0)
Pericardial tamponades requiring intervention	0 (0)	0 (0)
Postinterventional pericarditis or small pericardial effusion	4 (3.23)	2 (2.67)
Hemothorax	0 (0)	0 (0)
Severe air embolism with ST elevation	0 (0)	0 (0)
New-onset renal failure requiring dialysis	0 (0)	0 (0)
Vascular access complications requiring intervention	0 (0)	0 (0)
Vascular access complications without intervention	1 (0.8)	1 (1.33)
Acute hypersensitivity reactions to the contrast agent	0 (0)	0 (0)

Data given as *n* (%). Abbreviations: TIA, transient ischemic attack.

**Table 4:** Multivariable logistic regression for AF recurrence after PVI.

Variable	Adjusted OR	95% CI	*p*-Value
Coffee consumption (high vs. low)	0.47	0.23–0.95	0.036 *
Age (per year)	1.01	0.98–1.05	0.43
Persistent AF	2.46	1.06–6.33	0.046 *
LVEF (per 1% increase)	1.00	0.97–1.04	0.95
LA diameter (per 1 mm increase)	1.05	1.00–1.10	0.07

Data are presented as odds ratios (OR) with 95% confidence intervals (CI). Variables included in the model were age, type of atrial fibrillation, left ventricular ejection fraction, and left atrial diameter. * *p* < 0.05 was considered statistically significant. Abbreviations: AF, atrial fibrillation; LA, left atrium; LVEF, left ventricular ejection fraction; OR, odds ratio; CI, confidence interval.

## Data Availability

The data presented in this study are available on request from the corresponding authors.
